# A Novel One Health Approach concerning Yeast Present in the Oral Microbiome of the Endangered Rio Skate (*Rioraja agassizii*) from Southeastern Brazil

**DOI:** 10.3390/microorganisms11081969

**Published:** 2023-07-31

**Authors:** Manoel Marques Evangelista Oliveira, Amanda Pontes Lopes, Tatiane Nobre Pinto, Gisela Lara da Costa, Aristóteles Goes-Neto, Rachel Ann Hauser-Davis

**Affiliations:** 1 Laboratory of Taxonomy, Biochemistry and Bioprospecting of Fungi, Oswaldo Cruz Institute, Oswaldo Cruz Foundation, Rio de Janeiro 21040360, RJ, Brazil; 2 Laboratório de Avaliação e Promoção da Saúde Ambiental, Oswaldo Cruz Institute, Oswaldo Cruz Foundation, Rio de Janeiro 21040360, RJ, Brazil; 3 Institute of Biological Sciences, Federal University of Minas Gerais (UFMG), Belo Horizonte 30130100, MG, Brazilarigoesneto@icb.ufmg.br (A.G.-N.)

**Keywords:** yeast, *Rioja agassizii*, One Health, elasmobranchii

## Abstract

The current climate change scenario caused by anthropogenic activities has resulted in novel environmental pressures, increasing the occurrence and severity of fungal infections in the marine environment. Research on fungi in several taxonomic groups is widespread although not the case for elasmobranchs (sharks and rays). In this context, the aim of the present study was to screen the oral fungal microbiota present in artisanally captured *Rioraja agassizii*, a batoid that, although endangered, is highly fished and consumed worldwide. Oropharyngeal samples were obtained by swabbing and the samples were investigated using morphological and phenotypic methods by streaking on Sabouraud Dextrose Agar (SDA) and subculturing onto CHROMagar Candida (BD Difco) and CHROMagar Candida Plus (CHROMagar^TM^), as well as molecular techniques by amplification of the ITS1-5.8S-ITS2 ribosomal DNA region and a MALDI-TOF MS assessment. The findings indicated the presence of *Candida parapsilosis* (seven isolates), *Candida duobushaemulonii* (one isolate) and *Rhodotorula mucilaginosa* (three isolates), several of these reported for the first time in *Rioraja agassizii*. In addition, a 100% agreement between the MALDI-TOF results and partial ITS region sequencing was noted, demonstrating that the MALDI-TOF MS is a rapid and effective alternative for yeast identification in *Rioraja agassizii* isolates and potentially in other elasmobranch species. These findings highlight the need for further research to determine the potential impact on elasmobranch health, ecology, and commercial fisheries. Furthermore, this research is paramount in a One Health framework and may be employed to predict elasmobranch responses to an evolving ocean, keep healthy populations in check, monitor species, and assess the public health consequences of consuming these species.

## 1. Introduction

The effects of the current climate change scenario caused by anthropogenic activities [1] are creating novel environmental pressures, enough to increase the occurrence and severity of marine animal diseases [2], including fungal infections [3]. In this context, certain abiotic factors, such as salinity, light, temperature, sediment conditions, and chemical pollution, which are currently being altered at dramatic rates due to climate change effects, have been noted as significant drivers for marine fungal growth [4,5]. Even though the number of marine fungal pathogens has been estimated at over 10,000 species [6], their effects are still, nonetheless, largely understudied. Some assessments, however, have indicated their relevance to marine animal health, as they may indirectly affect marine ecology and directly affect the success of conservation efforts and economic viability of commercially important species [2]. The microbiome of several animals, such as fish, has, in fact, been significantly associated with environmental variations, including sea surface temperature, among others [7], indicating the importance of investigating marine microbiomes associated with climate change effects. However, the scarce assessments concerning fish have been mostly conducted on teleosts [8], despite the importance of cartilaginous (elasmobranch) fisheries worldwide, with rare assessments in elasmobranchs.

Most elasmobranchs, a group comprising rays and sharks, are threatened to some degree, mainly due to overfishing, chemical pollution, and habitat destruction [9], although climate change has also been pointed out as a significant threat to this group. The reason for this is multifold, including, but not limited to water current changes, altered environmental contaminant dynamics, alterations in physicochemical water properties and, finally, marine microbiota transformations. In this regard, elasmobranch microbiome research has intensified dramatically in recent years, albeit mostly for sharks, with a significant knowledge gap noted for rays and skates, even though these are more threatened than previously estimated, with 36.0% (*n* = 220 of 611) of known species now threatened compared to sharks (31.2%, *n* = 167 of 536) [10]. This type of assessment is important in a One Health framework, which recognizes the interconnection between people, animals, plants, and their shared environments, comprising a collaborative, multisectoral, and transdisciplinary approach at local, regional, national, and global levels [11]. This One Health approach, still very recent, draws attention to the links between biodiversity, which signal a healthy ecosystem, and human and animal health [11].

The One Health framework can be directly applied to environmental conservation, as it is also considered the basis for human health, since different anthropogenic activities lead to habitat degradation, resource overexploitation, and pollution, among many others [12]. Therefore, biodiversity losses are directly associated with the loss of economic, social, and environmental benefits, jointly called ecosystem services [13], leading to significant negative impacts on socioeconomic and cultural activities, and, consequently, Public Health. Concerning elasmobranch microbiome research, findings in this regard can be applied in a multidisciplinary manner, *i.e*., in predicting how sharks and rays respond to a changing ocean, in managing healthy populations under managed care and monitoring, and, finally, in a Public Health context, as most elasmobranchs consist of a cheap source of protein for humans worldwide. 

In this regard, the application of the holobiont concept, comprising hosts and all of their associated microorganisms, has increasingly become a valuable tool in several research fields [14], such as in biodiversity loss assessments and Public Health investigations. Shifts in symbiotic microbiota–host associations, have, for example, been proven as paramount in fish health maintenance as per Llewellyn et al. [15], with direct Public Health consequences, and recent studies have unveiled the significant role of fungi as emerging contributors to infectious diseases, with some, in fact, leading to severe population declines in certain fish species [16].

The elasmobranch *Rioraja agassizii* (Müller & Henle, 1841), commonly known as the Rio Skate, inhabits waters of up to 130 m in depth, and is distributed throughout the Atlantic coast of South America, from Brazil to southern Argentina. It is endemic on the coast of Rio de Janeiro, in southeastern Brazil [17], and even though it has been recently categorized as Vulnerable (VU) by the International Union for Conservation of Nature Red List, mainly due to its constant capture as bycatch in fisheries, no conservation measures have yet been implemented for this genus [18,19,20]. This is extremely worrying, as significant population reductions have been reported for this species and, in fact, the Rio skate is one of the most abundant elasmobranch species associated with fish landings in southeastern Brazil, and highly consumed by local human populations [21].

This clearly indicates that elasmobranch microbiome investigations are paramount both in an ecological context and in a Public Health framework. Therefore, this study aimed to carry out a yeast screening in the oral mucosa microbiota of *Rioraja agassizii* specimens sampled in Rio de Janeiro, southeastern Brazil. This is the first report of its kind for this skate species, and the obtained data represent a valuable fungal data baseline that may be applied to future conservation efforts. This is especially true under a climate change scenario, where altered oceanographic conditions are constantly leading to significant changes in the marine environment compared to previous conditions.

## 2. Material and Methods

### 2.1. Sampling

A total of six adult *Rioraja agassizii* specimens were captured on 18 May 2022 by artisanal fishers at Recreio, a neighborhood in the metropolitan region of the city of Rio de Janeiro, in the state of Rio de Janeiro, southeastern Brazil. The males (*n* = 2) weighed 300 and 355 g, with total lengths of 38.8 cm and 42.7 cm and disk widths of 25.1 and 27.3 cm, respectively, while the females (*n* = 4) weighed 650 g, 695 g, 735 g, and 995 g, with total lengths of 49.0 cm, 45.8 cm, 52.1 cm, and 52.2 cm and disk widths of 31.7 cm, 33.5 cm, 33.6 cm, and 32 cm, respectively. Only recently deceased animals were sampled, under SISBIO sampling license no. 77310-5. An ethical review or approval is not required for this study as an ethics committee authorization in Brazil is not required for the analyses of animal carcasses.

Oropharyngeal swabs (Olen, São Paulo, Brazil) were inserted into the posterior oropharynx, rubbed throughout the posterior pharynx and entire oral mucosa for 2 to 3 s and then placed into 5 mL tubes containing 2 mL of a 0.9% saline solution. The samples were then taken to the laboratory within 30 min of the samplings and kept at 4 °C until analysis.

### 2.2. Culture and Phenotypic Detection

Samples were streaked onto Sabouraud Dextrose Agar (SDA) and incubated at 30 °C for 48 h for morphological assessments. All samples presenting growth on the SDA medium were then subcultured onto CHROMagar Candida (BD Difco, Franklin, NJ, USA) and CHROMagar Candida Plus (CHROMagar^TM^, Paris, France) and colonies were interpreted according to the manufacturer’s instructions.

### 2.3. MALDI-TOF Assay

In addition to morphologic and phenotypic tests, obtained isolates were also identified using molecular tools, namely MALDI-TOF MS and ITS region sequencing [22]. Fungal identification at the species level by MALDI-TOF MS was carried out as previously described by Pinto et al. [22]. Briefly, 10^6^ yeast cells were transferred from the culture plate (c.a. 1 μg) to a 500 μL tube containing 20 µL of 70% formic acid in water (*v*/*v*) and mixed with 10 µL of acetonitrile. The samples (1 µL) were then spotted onto a stainless Bruker MALDI-TOF MS plate (Bruker Daltonics, Bremen, Germany) and covered with 1 µL of a α-cyano-4-hydroxycinnamic acid (CHCA, Fluka, Buchs, Switzerland) used as the matrix. Each sample was analyzed in triplicate. Samples were air-dried at room temperature prior to spectra acquisition. Results are expressed as log values ranging from 0 to 3, where values of 1.7 are generally used for reliable genus identification and score values of 2.0 indicate completely reliable species identification [23]. 

### 2.4. Molecular Approach

The extraction of genomic DNA from the yeast colonies grown on SDA was performed using Gentra^®^ Puregene^®^ Yeast and G+ Bacteria kits (Qiagen^®^, Germantown, MD, USA) according to the manufacturer’s recommendations. The amplification of the ITS1-5.8S-ITS2 ribosomal DNA region was performed in a final 50 µL volume containing 100 ng of DNA and 10 µL of each primer (Invitrogen^TM^, São Paulo, Brazil), ITS1 (5′ TCCGTAGGTGAACCTGCGG 3′), and ITS4 (5′TCCTCCGCTTATTGATATGC 3′) [24]. PCRs were performed employing a Veriti Applied Biosystems thermocycler at an annealing temperature of 58 °C. Automated sequencing was performed using the Fundação Oswaldo Cruz sequencing platform (PDTIS/FIOCRUZ, Rio de Janeiro, Brazil). The sequences were edited using CodonCode Aligner (Genes Code Corporation, Ann Arbor, MI, USA), and phylogenetic analyses were performed using the Blast software for comparison with sequences deposited in the NCBI/GenBank database.

Phylogenetic relationships were conducted using the MEGA X software version 10 (Tokyo, Japan) [25], determined using the Neighbor-Joining method [26]. The evolutionary distances were computed using the Maximum Composite Likelihood method [27]. Evolutionary analyses were conducted in the MEGA X software version 10 (Tokyo, Japan) [25]. 

## 3. Results 

**Figure 1:**
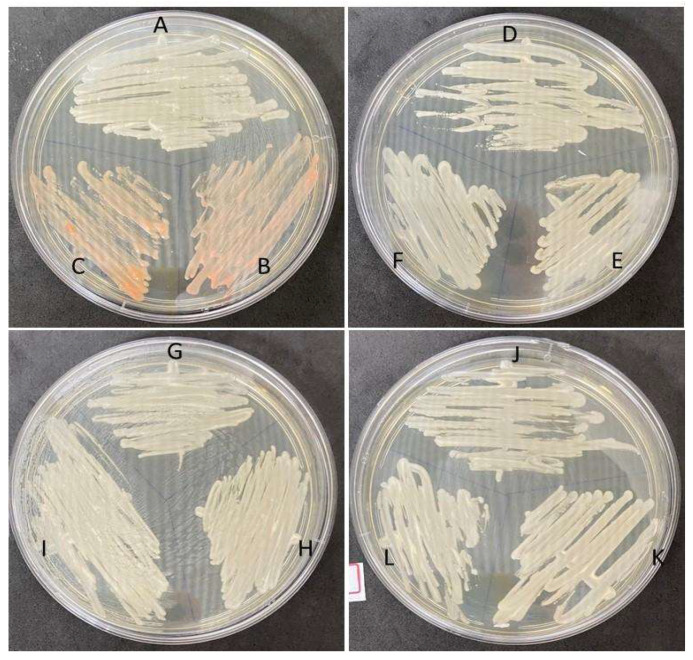
Isolate growth in SDA Medium (BD Difco) incubated at 30 °C for 48 h, (A) A87S1, (B) A84S2, (C) A86S2, (D) A84S1, (E) A84C1, (F) A86S1, (G) A87C1, (H) A88S1, (I) A89S1, (J) A85C2, (K) A85S1, (L) A85C1.

**Figure 2:**
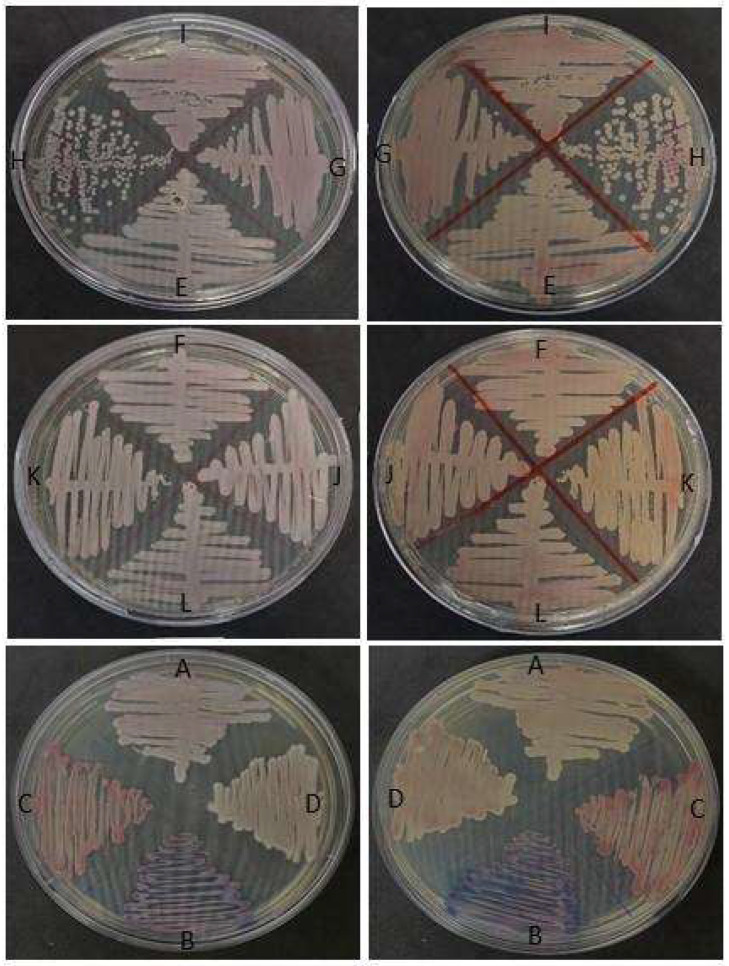
Growth of isolates in BD^TM^ CHROMagar^TM^ Candida Medium (BD Difco) incubated at 30 °C for 48 h, (A) A87S1, (B) A84S2, (C) A86S2, (D) A84S1, (E) A84C1, (F) A86S1, (G) A87C1, (H) A88S1, (I) A89S1, (J) A85C2, (K) A85S1, (L) A85C1.

Colony growths in the SDA medium used for fungal screening were observed for all of the animals included in this study (Figure 1), and the observed growths were then subcultured onto CHROMagar Candida (BD Difco) and CHROMagar Candida Plus (CHROMagar^TM^) (Figure 2). Colonies were interpreted according to the manufacturer’s instructions. 

White and orange colonies were observed in the SDA medium (Figure 1A), while white to pink and orange colonies were observed in both chromogenic media (Figure 1B,C), not allowing for a conclusive identification at the species level. 

Isolates were identified at the species level using the Bruker database, as *Candida parapsilosis* (seven isolates) and *Rhodotorula mucilaginosa* (three isolates) (Table 1), with a score range from 1.70 to 2.19. 

*Candida parapsilosis* and *Candida parapsilosis* were detected in the two male *Rioraja agassizii* specimens, while females presented, in addition to these two yeast species, *Rhodotorula* sp., *Candida krusei*, and *Candida krusei*. 

The amplification sequences of the ITS1-5.8S-ITS2 ribosomal DNA region were edited using the CodonCodeAligner 9.0.2. software and compared using a basic local alignment search tool with sequences available at the NCBI/GenBank database. The findings indicate 100% agreement with *Candida parapsilosis* (A87C1, A84S1, A85C2, A85C1, A84C1, A89S1, and A88S1), *Candida duobushaemulonis* (A86S1), and *Rhodotorula mucilaginosa* (A86S2, A84S2, and A87S1) for ITS sequences deposited in the GenBank. The ITS sequences of the isolates have been deposited in the GenBank under accession numbers OQ998993, OQ998994, OQ998995, OQ998996, OQ998997, OQ998998, OQ998999, OQ999000, OQ999001, OQ999047, OQ999048, and OQ999049. 

**Figure 3:**
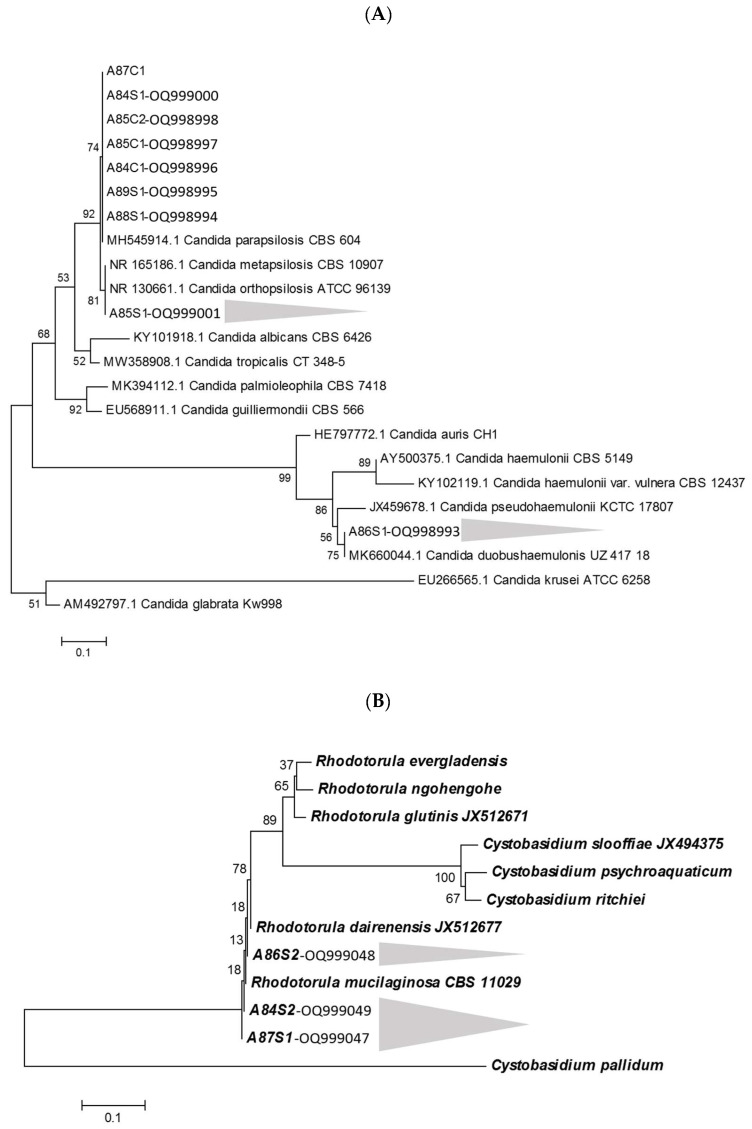
(**A**) Phylogenetic relationships between the isolates and reference *Candida* sp. strains inferred from ITS sequences by the Neighbor-Joining method [26]. The optimal tree is shown. The percentages of replicate trees in which the associated taxa clustered together in the bootstrap test (1000 replicates) are shown next to the branches [28]. The evolutionary distances were computed using the Maximum Composite Likelihood method [27] and are in the units of the number of base substitutions per site. This analysis involved 23 nucleotide sequences. A total of 229 positions were obtained in the final dataset. Evolutionary analyses were conducted in MEGA X [25]. (**B**) Phylogenetic relationships between the isolates and reference *Rhodotorula* sp. strains inferred from ITS sequences by Neighbor-Joining method [26]. The optimal tree is shown. The percentages of replicate trees in which the associated taxa clustered together in the bootstrap test (1000 replicates) are shown next to the branches [28]. The evolutionary distances were computed using the Maximum Composite Likelihood method [27] and are in the units of the number of base substitutions per site. This analysis involved 12 nucleotide sequences. A total of 443 positions were obtained in the final dataset. Evolutionary analyses were conducted in MEGA X [25].

Phylogenetic analyses were performed using MEGA X software, version 10 (Tokyo, Japan) [25]. Sequences corresponding to the ITS genes from *Candida* sp. and *Rhodotorula* sp. were obtained from the GenBank database (www.ncbi.nlm.nih.gov/genbank/) (Figure 3). All the samples were identified by MALDI-TOF MS and partial ITS region sequences, with a 100% agreement between both methodologies.

## 4. Discussion

Only some assessments on fungal diseases in elasmobranchs have been conducted to date. In this regard, one systematic review indicated that fungal infections are quite scarce in elasmobranchs, counting only 10 cases out of 1546 elasmobranch individuals when assessing the Northwest ZooPath database for cases recorded from 1994 to 2010 [29]. The recorded fungal infections included dermatitis (30%), hepatitis (30%), and branchitis (20%). A more recent systematic review [30] followed the same trend, retrieving only six fungal assessments in elasmobranchs, with most studies conducted on captive sharks from aquaria/oceanaria, and very scarce reports in rays from natural environments and aquaria/oceanaria. Three concerned the presence of *Fusarium solani*, two assessments reporting the presence of this fungus in the head and lateral line of *Sphyrna lewini* and one concerning the same fungus in the ventral pectoral fin of *Teaniura melanopsila* and the head and lateral line of *Sphyrna lewini*, another on *Exophiala* sp. on the head of *Cephaloscyllium ventriosum*, one on *Dasyatispora levantinae* in the skeletal musculature of *Dasyatis pastinaca*, one on *Paecilomyces lilacinus*, *Mucor circinelloides*, and *Exophiala pisciphila* in the liver, heart, kidney, spleen, and gills of *Sphyrna mokarran*, *Stegostoma fasciatum*, and one on *Fusarium keratoplasticum*, *Fusarium solani*, and *Metarhizium robertsii* in the head and lateral line of *Sphyrna lewini* and *Sphyrna tiburo*.

*Fusarium solani* has been pointed out for causing severe systemic mycosis in elasmobranchs [31,32,33], resulting in skin lesions characterized by ulcers and hemorrhage, and white and purulent exudates in cephalic canals and lateral lines, in some cases leading to animal death [32]. Other fungi have also been reported as infecting elasmobranchs, such as microsporidium in the muscle of common stingrays *Dasyatis pastinaca* (Diamant et al., 2010), *Paecilomyces lilacinus*, *Mucor circinelloides*, and *Exophiala pisciphila* in the Great hammerhead and the Zebra shark *Stegostoma fasciatum* [34], leading to death, and *Exophiala* sp. in a Swell shark *Cephaloscyllium ventriosum* [35], leading to abnormal swimming behavior. 

Antifungal drugs directly influence the composition of marine bacterial communities [36]. In this regard, fungi have been noted as increasingly developing resistance to drugs in the same way that bacteria develop resistance to antibiotics [37]. This has become a global concern, as the incidence of fungal infections has increased alarmingly in the last decades, with high mortality and morbidity rates due to poor diagnosis, drug-resistance, and lack of effective antifungal therapy, as well as the fact that some fungi are able to form biofilms with high resistance to most conventional antifungals [38]. This leads to Public Health concerns regarding antifungal resistance, as sharks and rays are commonly consumed worldwide, comprising a cheap source of protein for millions of people [39,40]. Brazil is, in fact, one of the most important shark meat consumers worldwide, although most sharks are sold under other names, such as “*cação*”, hindering correct identification by consumers [41]. To further aggravate matters, a chronic lack of species-specific monitoring and identification is noted throughout the country [41], making consumers even more vulnerable to buying and consuming both threatened and contaminated elasmobranchs. Thus, this study may also contribute towards indirect elasmobranch conservation, as revealing significant human health risks associated to the consumption of these animals due to pathogenic fungi may halt or, at least, reduce their consumption, as demonstrated for other taxonomic groups [42,43].

In addition, elasmobranchs are routinely handled by fishers who rarely wear protective equipment and are, thus, exposed to pathogens, worsened by the fact that some sharks and rays exhibit rough skin and spines, leading to scratches and open wounds in these vulnerable workers. They are also sold in unsanitary conditions in many regions worldwide and encounter other seafood sold to consumers, such as mussels, crustaceans, and teleost fish, during and after processing. The emergence of fungal resistance along the food chain is an important global Public Health problem, as resistant fungi and antifungal-resistant genes can spread at each stage of the food production chain, *i.e*., harvesting, transportation, and storage [44,45]. Indeed, the issue of fungal resistance has gained prominence globally. This includes the emergence of new strains of previously vulnerable fungi that have developed resistance, as well as the emergence of new species impervious to multiple antifungal medications, such as *Candida auris*, a distinct *Candida* species from that reported herein (see [46] for details), although belonging to the same genus, further indicating the need for deeper assessments in this regard. 

It is also important to note that the elasmobranch analyzed herein was a skate, with benthic habits, and thus highly vulnerable to pathogen exposure, as marine sediments are the ultimate sink for several contaminants [47], including metals, organic compounds and pathogens and microplastics. Specifically concerning the latter, microplastic contamination has increasingly become a worldwide problem due to their poor management and disposal, exacerbating pathogenic exposure potential, as several studies have reported microplastics as vectors for pathogens, including fungi [48]. 

*Candida parapsilosis* exhibits high zoonotic potential, although it mostly infects immunocompromised hosts. In humans, *C. parapsilosis* has been associated with disease endocarditis, meningitis, septicemia, peritonitis, arthritis, endophthalmitis, keratitis, otitis, cystitis, and skin infections [49,50], and has been isolated from cats and cockatiels after a history of eating marine foods [51,52,53]. In fish, *Candida parapsilosis* has been reported as the causative agent of a disseminated mycotic granulomatous lesion leading to death in a marine Okhotsk snailfish (*Liparis ochotensis*) kept at an aquarium. Reports of this fungi in aquatic organisms, however, are scarce. Tartor and colleagues [54] demonstrated the isolation of yeasts in dead freshwater fish in Egypt, identifying *Candida* and *Rhodotorula* genera species employing MALDI TOF MS. *Rhodotorula* sp. has also been previously described as being isolated in freshwater fish in the Odra River estuary in the Baltic Sea, and *Rhodotorula mucilaginosa* has been described as commensal in the gastrointestinal microbiota of these fish [55]. However, fungi research in cartilaginous fish is still scarce.

The isolates obtained in our study yielded colonies which stained diversely in the chromogenic CHROMagar Candida and CHROMagar Candida Plus media. All samples generated one color pattern, demonstrating no mixed colonies. Thus, these chromogenic media seem not to be the best choice for yeast screening from the oral mucosa microbiota obtained from *Rioraja agassizii* specimens. Thus, from an identification perspective, the CHROMagar Candida medium can be clearly misleading, as this medium is not designed to discriminate new emerging species. The MALDI-TOF MS, however, presented a 100% agreement with the obtained partial ITS region sequences. 

The MALDI-TOF MS technique has been recently introduced in many clinical microbiology laboratories, comprising a fast and powerful tool capable of identifying fungal species belonging to different fungal genera [56,57,58,59]. Flórez-Muñoz and collaborators [60], for example, reported that the use of MALDI-TOF MS in clinical settings is a reliable, fast, and cost-effective fungal identification system, although indicating that an associated and accurate database is essential, while Matos and collaborators [61] specified the absence of fast methodologies for the diagnosis of certain fungal diseases, such as sporotrichosis, an endemic mycose, demonstrating that the MALDI-TOF MS is adequate in this regard when a reliable spectral database is available. Furthermore, one study by Chen et al. [62] reported that the MALDI-TOF MS method is superior in identifying yeast and mycobacteria isolates compared to gene sequencing, as it was able to differentiate *Mycobacterium* sp. clinical species, despite the fact that no differentiation between *Mycobacterium abscessus* and *Mycobacterium chelonae* was found using 16S rRNA gene sequencing. The authors, thus, proved that the MALDI-TOF MS method is suitable as a first-line test for the identification of yeast and mycobacteria, with very short turnaround times. In another study, Stravou et al. [63] compiled numerous studies reporting accurate yeast identification, characterizing multiple *Candida* complexes and *Saccharomycotina* fungi using MALDI-TOF MS complemented by computational identification methods, such as global data bank and sequence alignment searches. In the present study, the original MALDI-TOF MS database used for yeast identification already contained reference spectra for a variety of fungal species, *i.e*., *C. parapsilosis*, *C. duobushaemulonis*, and *Rhodotorula mucilaginosa*. This reinforces that the MALDI-TOF MS method can comprise a quick and effective alternative to classical and phenotypic methods for fungal identification, including the Chromoagar *Candida* medium, in identifying yeast isolates from *Rioraja agassizii*, potentially extendable to other elasmobranch species, although further assessments are required in this regard.

Concerning study limitations, the relatively small sample size of six individuals should be noted. Although several elasmobranchs display sex-based sexual segregation during certain life stages, the low number of sampled *Rioraja agassizii* specimens also precludes any hypotheses on sex-based yeast colonization differences. Furthermore, although the samples were obtained during the dry season in Rio de Janeiro, it is unknown if seasonality would be a significant factor in the fugal colonization of *Rioraja agassizii*. We do not believe in any bias due to artisanal capture to be in place, as these animals are caught in their regular habitats by gill nets and immediately brought to land for marketing.

## 5. Conclusions

Several fungi detected in the oral mucosa microbiota of healthy *Rioraja agassizii* specimens are reported herein for the first time, suggesting this species as a potential sentinel for emerging fungal pathogens. Ecological effects of the determined fungi, however, are still unknown. Additionally, this species may also play a role in the establishment of an infectious process in other taxonomic groups, including in humans. Elasmobranch associations to fungal pathogens are, however, still understudied, and fungal biodiversity, prevalence, and physiological effect assessments are extremely scarce, even though they are clearly paramount in the face of climate change effects, elasmobranch vulnerability, and the zoonotic potential of many fungi species. This clearly indicates the need for further biomonitoring studies, both in *Rioraja agassizii* specimens and in other elasmobranch species routinely consumed by humans to further evaluate fungal pathogens in elasmobranchs through a One Health framework. Thus, future studies should aim to assess a higher number of specimens, both males and females, and potential seasonality effects, as well as screen resistant genes encoded by fungi to further evaluate their public health significance concerning antifungal resistance. Furthermore, it is important to note that conventional identification methods require several biochemical and phenotypic tests, which can lead to lengthy and yet, insufficient, precision to discriminate yeast species, paving the way for the use of MALDI-TOF MS as an adequate and powerful technique for fungal biomonitoring. 

## Figures and Tables

**Table 1:** Identified fungal isolates obtained from *Rioraja agassizii* oropharyngeal swabs at the species level using the Bruker database. The sex and identification number of the *Rioraja agassizii* specimens are also indicated.

Sample	*Rioraja agassizii* Individual	Methodologies		
		Chromoagar Candida	ITS Sequencing Partial	MALDI-TOF MS (Species/Score)
A84C1	Male 1	*Candida parapsilosis*	*Candida parapsilosis*	*Candida parapsilosis—* **Score 1.77**
A84S1	Male 1	*Candida parapsilosis*	*Candida parapsilosis*	*Candida parapsilosis—* **Score 1.78**
A84S2	Male 1	*Candida tropicalis*	*Rhodotorula mucilaginosa*	*Rhodotorula mucilaginosa—* **Score 1.70**
A85C1	Male 2	*Candida parapsilosis*	*Candida parapsilosis*	*Candida parapsilosis—* **Score 1.76**
A85C2	Male 2	*Candida parapsilosis*	*Candida parapsilosis*	*Candida parapsilosis—* **Score 2.19**
A85S1	Male 2	*Candida parapsilosis*	*Candida orthopsilosis*	*Candida orthopsilosis—* **Score 1.97**
A86S1	Female 1	*Candida parapsilosis*	*Candida duobushaemulonis*	*Candida duobushaemulonis—* **Score 1.76**
A86S2	Female 1	*Rhodotorula* sp.	*Rhodotorula mucilaginosa*	*Rhodotorula mucilaginosa—* **Score 1.73**
A87C1	Female 2	*Candida krusei*	*Candida parapsilosis*	*Candida parapsilosis—* **Score 1.73**
A87S1	Female 2	*Candida parapsilosis*	*Rhodotorula mucilaginosa*	*Rhodotorula mucilaginosa—* **Score 1.70**
A88S1	Female 3	*Candida parapsilosis*	*Candida parapsilosis*	*Candida parapsilosis—* **Score 1.72**
A89S1	Female 4	*Candida krusei*	*Candida parapsilosis*	*Candida parapsilosis—* **Score 2.19**

## Data Availability

The data presented in this study are available on request from the corresponding authors.
